# Microglial Drug Targets in AD: Opportunities and Challenges in Drug Discovery and Development

**DOI:** 10.3389/fphar.2019.00840

**Published:** 2019-08-23

**Authors:** Knut Biber, Anindya Bhattacharya, Brian M. Campbell, Justin R. Piro, Michael Rohe, Roland G.W. Staal, Robert V. Talanian, Thomas Möller

**Affiliations:** ^1^AbbVie Deutschland GmbH & Co. KG, Neuroscience Research, Ludwigshafen, Germany; ^2^Janssen Research & Development LLC, San Diego, CA, United States; ^3^Sage Therapeutics, Cambridge, MA, United States; ^4^AbbVie Foundational Neuroscience Center, Cambridge, MA, United States; ^5^Paracelsus Neuroscience, Metuchen, NJ, United States

**Keywords:** drug target, microglia, target identification, target validation, screening cascade

## Abstract

Alzheimer’s disease (AD) is a large and increasing unmet medical need with no disease-modifying treatment currently available. Genetic evidence from genome-wide association studies (GWASs) and gene network analysis has clearly revealed a key role of the innate immune system in the brain, of which microglia are the most important element. Single-nucleotide polymorphisms (SNPs) in genes predominantly expressed in microglia have been associated with altered risk of developing AD. Furthermore, microglia-specific pathways are affected on the messenger RNA (mRNA) expression level in post-mortem AD tissue and in mouse models of AD. Together these findings have increased the interest in microglia biology, and numerous scientific reports have proposed microglial molecules and pathways as drug targets for AD. Target identification and validation are generally the first steps in drug discovery. Both target validation and drug lead identification for central nervous system (CNS) targets and diseases entail additional significant obstacles compared to peripheral targets and diseases. This makes CNS drug discovery, even with well-validated targets, challenging. In this article, we will illustrate the special challenges of AD drug discovery by discussing the viability/practicality of possible microglia drug targets including cluster of differentiation 33 (CD33), K_Ca_3.1, kynurenines, ionotropic P2 receptor 7 (P2X7), programmed death-1 (PD-1), Toll-like receptors (TLRs), and triggering receptor expressed in myeloid cells 2 (TREM2).

## Highlights:

- Glial cell–based targets receive increased attention in drug development.
- What makes a good drug target?
- Steps and pitfalls for preclinical drug development.
- Specific consideration for putative microglial drug targets in Alzheimer’s disease.

## Introduction

Microglial reactivity has long been recognized as a pathological hallmark of a wide variety of neurological diseases. While morphological changes of microglia were initially interpreted as a reactive response to neuronal damage, growing evidence suggests that these changes are not merely “reactive” but indicate that glial pathology contributes to disease progression (Raj et al., 2014a; Verkhratsky et al., 2014; Ben Haim et al., 2015; Bergles and Richardson, 2015; Biber et al., 2015; Heneka et al., 2015; Heppner et al., 2015; Jain et al., 2015; Ransohoff and El Khoury, 2015; Robel and Sontheimer, 2015; Zuchero and Barres, 2015; Osborn et al., 2016). Especially with regard to Alzheimer’s disease (AD), numerous reviews cover various aspects of microglial biology in the context of disease (see for example: (Guedes et al., 2018; Hansen et al., 2018; Kinney et al., 2018; Shi and Holtzman, 2018; Thei et al., 2018; Ulland and Colonna, 2018; Henstridge et al., 2019). Using the search term “microglia Alzheimer,” PubMed lists close to 100 reviews that were published in the last 2 years (Jan 2017–Jan 2019). It is not the purpose of the current review to discuss the potential role of microglia in AD, but to focus on the challenges and practical aspects of microglial proteins or pathways as potential drug targets.

A considerable set of genetic risk factors for AD are predominantly expressed in microglia (Wes et al., 2016; Henstridge et al., 2019). Thus, it is very clear from human genetics that regardless of being “reactive,” “active,” “dystrophic,” “senescent,” “dysfunctional,” or “disease associated,” microglial involvement in AD offers a hitherto unexplored intervention point. Many publications concerning microglia in AD include conclusions like: “The presented data here suggest that protein ‘XYZ’ can be considered as a drug target for AD.” As there are some common misunderstandings between colleagues focused on biology vs. drug discovery regarding the definition of a target, we first aim to clarify what it takes to consider a microglial molecule suitable for a drug discovery program. We will then review some commonly suggested microglial targets and pathways, i.e., cluster of differentiation 33 (CD33), K_Ca_3.1, kynurenines (KYN), ionotropic P2 receptor 7 (P2X7), programmed death-1 (PD-1), Toll-like receptors (TLRs), and triggering receptor expressed in myeloid cells 2 (TREM2), discussing their potential role in AD and their respective challenges in regard to drug discovery.

## Target Identification

**Figure 1:**
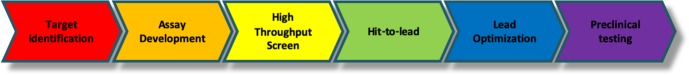
Schematic representation of the preclinical drug discovery process. The preclinical drug discovery process can generally be divided into six unique steps from target identification to preclinical testing.

In general, preclinical drug discovery can be staged as follows: target identification, in which the validity of a target for modulating a disease process and the initial chemical matter along with the means of characterizing them are established; lead optimization, in which target validity is further strengthened while identifying candidate molecules with the full range of necessary drug-like properties; and finally, preclinical testing for safety and efficacy. Apart from target identification, we will not discuss the other stages of preclinical drug development (**Figure 1**). The interested reader is referred to (Möller and Boddeke, 2016) for a detailed description of the whole process.

For potential targets, it is of utmost importance to demonstrate relevancy to the disease and that modulating the target would result in therapeutic benefit with an acceptable safety margin. Evidence for a role of a candidate target in disease might include i) altered expression in disease, ii) integral relationship to disease pathophysiology, iii) genetic association with disease, iv) having a mechanistic link to disease etiology, and v) demonstration of a beneficial effect when modulated in a disease-relevant *in vitro* or (preferably) *in vivo* model.

Unfortunately, generating expression data for the human central nervous system (CNS) is extremely difficult. While for peripheral diseases biopsies, or blood draws are routine, for the CNS, most parameters can only be derived from post-mortem brain tissue that at best represents end-stage disease, or by limited imaging methods, making it difficult to observe changes in earlier and therapeutically more relevant disease states. Non-human models can also provide insight, but with significant translational uncertainties.

A strong genetic association of a microglial molecule with AD, for example, CD33, complement receptor 1 (CR1), phospholipase C gamma 2 (PLCγ2), and TREM2 (Wes et al., 2016; Henstridge et al., 2019), also suggests promise as a drug target. If such evidence can further be strengthened by a mechanistic link to a disease model by *in vitro* or *in vivo* genetic or pharmacologic intervention studies [e.g., knocking out CD33 (Bradshaw et al., 2013)], that would serve to increase confidence in the target.

## What Makes a Good (Microglial) Target?

### Drugability

Besides linking a target to disease, there are other more pragmatic considerations for selecting a target. Admittedly, “drugable” is not a well-defined term; it usually includes chemical tractability with a ligand (small molecule or antibody) but can also refer more broadly to the properties of such ligands, e.g., pharmacokinetics, distribution, metabolism (and the pharmacologic properties of significant metabolites), pharmaceutical properties including chemical stability, and manufacturability.

Traditionally, a target would be considered chemically tractable if a small molecule or antibody can bind with high affinity and specificity and induce the desired biological effect. For example, G-protein coupled receptors (GPCRs) are generally considered chemically tractable; more than 30% of currently marketed drugs target GPCRs (Ma and Zemmel, 2002; Hauser et al., 2017). Ion channels or enzymes are also considered tractable as those targets often include structural features that permit potent and specific ligand binding, with consequent direct inhibition of conductance or catalytic activity. Blocking protein–protein interactions is generally more difficult; however, recent advances have challenged this view, and modulation of protein–protein interactions by small molecules is now an area of active research and will increase the number of targets considered drugable (Higueruelo et al., 2013). Furthermore, blocking protein–protein interactions can often be accomplished with therapeutic antibodies, and several innovative approaches have been devised to improve antibody penetration into the brain (Watts and Dennis, 2013; Salameh and Banks, 2014; Pardridge, 2015). Despite these recent advancements, developing therapeutics for the CNS remains challenging, and while there are many biologically interesting molecules related to microglia, only a small subset of them might be considered drugable.

### Specificity

It is not merely enough to generate a potent pharmacological agent against a drug target; it is also of utmost importance that the molecule is selectively modulating only this one target to avoid off-target effects that may hamper the interpretation of experimental data. Unfortunately, many so-called “reference compounds,” such as commercially available inhibitors, are not as specific as advertised by the suppliers (Frye, 2010). A group of 50 scientists from different life science disciplines have covered this topic in detail in Commentary in *Nature Chemical Biology* (Arrowsmith et al., 2015). This lack of specificity makes interpretation of results gained with many compounds virtually impossible. A prominent example in microglia biology (although not directly related to a single target) is minocycline, which often is referred to as a “microglia inhibitor” (Möller et al., 2016).

### Safety

Arguably the most important criterion for target selection, however, is safety. During clinical development, many drugs fail, not due to the lack of efficacy but due to adverse safety events inadequately predicted by preclinical studies. Toxicity might not only arise from modulating the target itself, meaning that the therapeutic window is small, but that there could be off-target effects of a drug, or that toxicity may also arise from a metabolite of the drug that itself is toxic. It is therefore important to select targets with the highest likelihood of being safe as early as possible. A common question is how broadly a target is expressed. Localized expression preferentially in the target cell and/or tissue is considered advantageous as there is less likelihood for adverse effects unrelated to the desired mechanism of action. Unfortunately, there are very few potential targets restricted to microglia as most potential targets are also expressed in peripheral myeloid cells, so effects on the peripheral immune system are a persistent concern. Safety concerns, whether based on side effects of a development compound, a target knockout phenotype, or suspected essential physiological roles of the target, need to be addressed as early as possible by experiments.

Taken together, there is a whole range of drugability considerations that must be met to make a target practical for drug discovery. There are few targets in microglia that are often discussed in the literature to be of value in AD. Here we will discuss the potential and challenges around these targets when it comes down to drugability aspects.

## Currently Proposed Microglial Drug Targets

### Proposed Role of CD33 in AD

In 2011, large-scale genome-wide association studies (GWASs) identified CD33, a member of the sialic acid–binding immunoglobulin-like lectins (siglecs) family, as a genetic locus associated with the risk to develop AD (Hollingworth et al., 2011; Naj et al., 2011). This association with AD risk has been confirmed in a variety of subsequent genetic studies in different ethnic groups, with odds ratios between 0.7 and 0.9; thus, the CD33 rs3865444A allele protects from the development of AD [for recent meta-analysis, see (Jiang et al., 2018)]. It was furthermore described that the protective single-nucleotide polymorphism (SNP) rs3865444A is in linkage disequilibrium with rs12459419T, which causes increased splicing of exon2, and therefore deletes the sialic acid–binding site of CD33. The protective SNP therefore leads to increased expression of a truncated, non-functional version of the receptor, called d2-CD33 or CD33m (Bradshaw et al., 2013; Malik et al., 2013; Raj et al., 2014b). A reduction in full-length CD33 expression was observed in myeloid cells of CD33 rs3865444A allele carriers, resulting in an overall reduced CD33 function (Bradshaw et al., 2013; Raj et al., 2014b).

CD33 signaling in humans depends on two intracellular inhibitory domains, one immunoreceptor tyrosine-based inhibitory motif (ITIM), and one ITIM-like domain. CD33 is therefore considered to be an inhibitory immune receptor. Indeed, when activated CD33 becomes tyrosine-phosphorylated by Src kinases, which subsequently recruit Src homology-2 domain (SH2)–containing tyrosine phosphatases (SHP1/2), this results in the dephosphorylation and inhibition of signaling cascades (see for review Macauley et al., 2014). CD33 is expressed in myeloid cells, and its signaling negatively regulates the function of these cells. Accordingly, knockdown of CD33 in human monocytes leads to spontaneous release of interleukin 1 beta (IL-1β), Tumor necrosis factor alpha (TNFα), and IL-8 (Lajaunias et al., 2005). In monocytes from carriers of the CD33 rs3865444A allele (decreased CD33 function), an increased uptake capacity of amyloid beta(1–42) (Aβ_1–42_) was described (Bradshaw et al., 2013).

**Figure 2:**
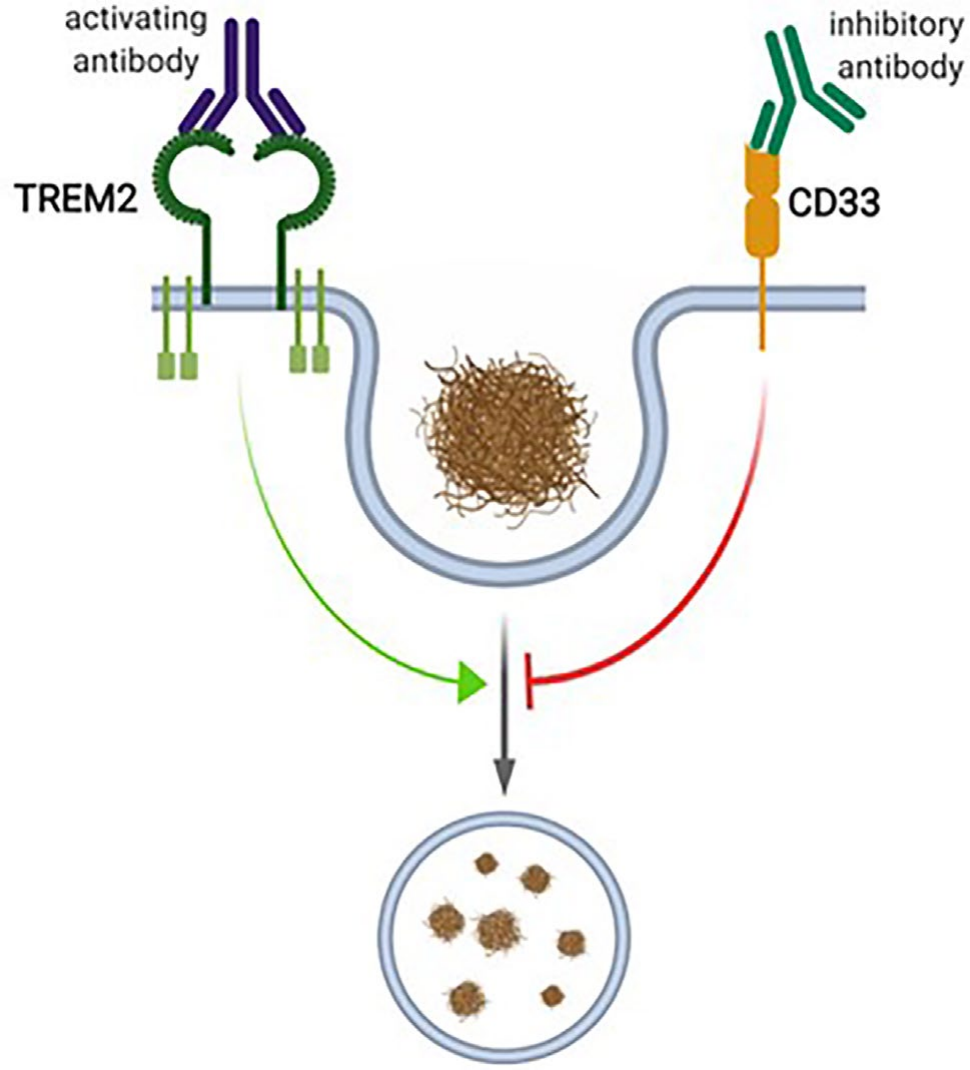
The proposed interplay between CD33 and TREM2 in microglial phagocytosis. CD33 and TREM2 have been proposed to be inhibitors or activators of microglial phagocytosis, respectively. For TREM2, activating antibodies, and for CD33, inhibitory antibodies, have been proposed as therapeutic interventions. Figure created with Biorender.com

In the brain, CD33 is specifically expressed in microglia (Bradshaw et al., 2013; Griciuc et al., 2013; Malik et al., 2013; Walker et al., 2015), where an inverse relationship between CD33 expression and Aβ uptake capacity was observed (Griciuc et al., 2013). Accordingly, in the cortex of AD patients, a positive correlation of microglial CD33 expression and amyloid pathology was described, indicating that increased CD33 expression in microglia promotes plaque pathology (Griciuc et al., 2013). CD33-deficient Amyloid precursor protein (APP)/Presenilin 1 (PS1) mice showed reduced amyloid pathology compared to age-matched wildtype (WT) controls, supporting the idea that reduced microglial CD33 function is beneficial with respect to amyloid pathology (Griciuc et al., 2013). Given this variety of data concerning the role of CD33 as regulator of microglial Aβ uptake capacity, this siglec receptor is currently considered as drug target in AD. It is considered that inhibition of CD33 function in microglia would increase their amyloid uptake capacity, which in turn would be beneficial in AD **Figure 2** (Heneka et al., 2015).

#### Target Challenges Around CD33

There are many challenges with respect to CD33 as a drug target in AD. In general, finding ligands for siglecs is difficult, as glycan binding often is of relatively low affinity and relies on multivalency to achieve adequate affinity (Varki, 2009). There is evidence that CD33 binds α2.3 sialic acid and α2.6 sialic acid; the corresponding glycosylated proteins that are decorated with these sialic acids, however, have not been identified (Linnartz-Gerlach et al., 2014; Macauley et al., 2014). As CD33 is the shortest of all known siglecs, it most likely is activated in a cis-manner (O’Reilly and Paulson, 2009; Linnartz-Gerlach et al., 2014; Macauley et al., 2014), meaning that the sialylated glycoproteins that activate CD33 would also be expressed in microglia. One report provides data that soluble sialyllactosamine in the mM range might activate CD33 in de-sialylated monocytes (Lajaunias et al., 2005). This finding awaits independent confirmation. Apart from our very limited understanding around natural ligands for CD33, functional tool compounds for CD33 have not yet been described. The only published molecule displaying a reasonable binding at CD33 is cpd22, which displaces bead-coupled α2.6 sialic acid with an half maximal effective concentration (EC_50_) of about 10 µM (Rillahan et al., 2014). It therefore is suggested that cpd22 binds CD33 at the sialic acid–binding domain; whether this binding affects CD33 signaling is not understood. Thus, it is not clear whether cpd22 might function as an agonist or antagonist for CD33. The lack of ligands or tool compounds seriously hampers the development of functional analysis of CD33 in cellular assays, making the development of functional inhibitors for CD33 difficult.

Therapeutic inhibitors of CD33 are hypothesized to increase phagocytic properties in microglia; from a drug development perspective, they might also be envisaged to inhibit the CD33 signaling cascade. It is not yet understood how recruitment of SHP1 leads to inhibition of cellular phagocytosis in CD33-expressing myeloid cells (Linnartz-Gerlach et al., 2014).

Another challenge concerns the large species difference of CD33. Mouse and human CD33 differ not only in the extracellular domain but also in the transmembrane domain as well as intracellularly. In contrast to human CD33, mouse CD33 only has one ITIM-like domain but lacks the ITIM domain (Cao and Crocker, 2011). Moreover, in the transmembrane region of mouse CD33, a positively charged residue is present, which might enable mouse CD33 to recruit immunoreceptor tyrosine-based activation motif (ITAM)-motif–containing adapter proteins like DNAX activating protein of 12 kDa (DAP12) (Linnartz-Gerlach et al., 2014). These data suggest that mouse CD33 can act as an activating receptor instead of being inhibitory, a hypothesis that awaits experimental evidence. In cultured microglia from CD33KO mice, it was shown that CD33 negatively couples to phagocytosis (Griciuc et al., 2013). Whether this is due to ITIM-like signaling or recruitment of ITAM-motif–containing adapter proteins is currently an open question. Potential drugs that inhibit CD33 function, however, need to be tested in animal models. Whether or not the mouse would be an adequate model system to do so is questionable given the species differences and the limited knowledge on CD33 function in mouse cells.

Taken together, despite the genetic association between CD33 and AD development and the straightforward hypothesis of how reduced CD33 function might protect from AD, CD33 remains a target with its unique set of challenges.

### Proposed Role of K_Ca_3.1 in AD

K_Ca_3.1 is an intermediate-conductance potassium channel that is activated when intracellular calcium is increased. The Ca^2+^ binds to calmodulin, which is constitutively bound to the calmodulin binding domain of the channel, resulting in the opening of the K^+^ channel (K_d_ ∼ 300 nM) (Xia et al., 1998; Fanger et al., 1999; Bouhy et al., 2011). The K_Ca_3.1-mediated K^+^ efflux augments the driving force for further Ca^2+^ influx *via* Ca^2+^ release-activated calcium (CRAC) channels and subsequent refilling of intracellular calcium stores. Thus, one of the primary functions of K_Ca_3.1 is the augmentation of Ca^2+^ signaling (Feske et al., 2012). Expression of K_Ca_3.1 in the CNS and its controversies have been reviewed (Dale et al., 2016). Here we will focus on microglia, as the expression of K_Ca_3.1 was established using well-validated antibodies and demonstration of voltage-independent K_Ca_3.1-like Ca^2+^-activated K^+^ currents.

Voltage-independent K_Ca_3.1-like Ca^2+^-activated K^+^ currents in cultured murine microglia were first reported by Eder et al. (Eder et al., 1997). Khanna et al. subsequently demonstrated the presence of K_Ca_3.1 mRNA in cultured rat and mouse microglia (Khanna et al., 2001). The conclusion that microglia express K_Ca_3.1 was supported by pharmacological studies that demonstrated that charybdotoxin and clotrimazole could inhibit the voltage-independent K_Ca_3.1-like Ca^2+^-activated K^+^ currents (Schilling et al., 2002; Schilling and Eder, 2004). There is further evidence that charybdotoxin and clotrimazole prevent the induced microglial oxidative burst (Khanna et al., 2001) and microglial migration (Schilling and Eder, 2004). The K_Ca_3.1 inhibitor TRAM-34 reduced lipopolysaccharide (LPS)-induced microglial neurotoxicity (Kaushal et al., 2007), and inhibition of K_Ca_3.1 using senicapoc (see below) blocked the LPS-induced IL-1β and Nitric oxide (NO) production by microglia (Staal et al., 2017a).

Early *in vivo* studies using antibodies validated in K_Ca_3.1 WT and knock out (KO) mice did not provide any evidence of K_Ca_3.1 expression on any cells in the mouse or human brain (including juvenile, adult, and aged mice) (Schilling and Eder, 2004; Schilling and Eder, 2007). However, studies using antibodies validated in this manner demonstrated K_Ca_3.1 expression on CD68-positive cells (microglia or macrophages) upon injury to the CNS as induced by ischemic injury (Chen et al., 2011), suggesting that K_Ca_3.1 is expressed by microglia only in response to injury. The study by Chen et al. also demonstrated the therapeutic efficacy of inhibiting K_Ca_3.1 (using TRAM-34) in ischemic injury, a finding they validated using K_Ca_3.1-null mice (Chen et al., 2011; Chen et al., 2015). Many other studies have since demonstrated the therapeutic efficacy of K_Ca_3.1 inhibition in other models of neurological injury and disease in which microglia are believed to play a significant role, including experimental autoimmune encephalomyelitis, traumatic brain injury, spinal cord injury, optic nerve transection, glioblastoma multiforme, neuropathic pain, and AD (Dale et al., 2016; Staal et al., 2017b).

Taken together, there is convincing evidence for the expression of K_Ca_3.1 in cultured rodent microglia. While the details of regulation of K_Ca_3.1 await confirmation with more specific tool compounds, the available data suggest a role for K_Ca_3.1 at the very least in microglia reactive oxygen species (ROS) production and signaling.

#### Challenges in K_Ca_3.1 Inhibition as Therapeutic Intervention in AD

The presence of “activated” microglia identified by expression of markers of microglial activation [major histocompatibility complex II (MHCII), Iba1] or an activated morphology (enlarged cell bodies and shorter, thicker processes) in brains of AD is well established. The observation that microglial “activation” can occur prior to plaque deposition and neurofibrillary tangle formation, and correlates with cognitive deficits, is consistent with the theory that microglial activation occurs at the earliest stages of disease (perhaps even before disease onset) and is somehow tied to its progression (Okello et al., 2009a; Okello et al., 2009b; Sastre et al., 2011). Maezawa et al. demonstrated that TRAM-34 blocks Aβ-induced proliferation of microglia, p38 Mitogen-activated protein kinase (MAPK) phosphorylation, nuclear factor kappa b (NF-κb) activation, and NO generation in primary microglia cultures (Maezawa et al., 2012). Furthermore, TRAM-34 inhibited neurotoxic effects of Aβ oligomers in mixed microglia–neuron cultures and in organotypic hippocampal slices by decreasing microglial activation and partially preventing synaptic loss (Maezawa et al., 2011).

Senicapoc is a potent and selective inhibitor of K_Ca_3.1 developed for the treatment of sickle cell anemia [half maximal inhibitory concentration (IC_50_) = 11 nM; screening of 57 targets yielded no hits below 1 µM other than K_Ca_3.1] (Ataga et al., 2006; Ataga et al., 2008; Ataga and Stocker, 2009; Dale et al., 2016; Staal et al., 2017a). In a recent and comprehensive study, Maezawa and colleagues demonstrated that K_Ca_3.1 is functionally upregulated in microglia in brains from 5xFAD mice as well as AD patients (Jin et al., In Press). The authors demonstrate that Aβ oligomers impair long-term potentiation (LTP) and that this effect is reversed by the K_Ca_3.1 inhibitor TRAM-34 as well as senicapoc in hippocampal slices or *in vivo*. It was furthermore demonstrated in this paper that senicapoc can reduce the pro-inflammatory effects as well as rescue the hippocampal LTP affected by Aβ oligomer (Jin et al., In Press). Senicapoc was then tested in the 5xFAD mice, starting at 6 months of age and continuing for three months. Results show that treatment with senicapoc reduced the amyloid load as well as neuroinflammation while enhancing neuronal plasticity compared to vehicle-treated mice (Jin et al., In Press). Thus, the study provides a strong rationale for repurposing senicapoc for the treatment of AD.

In fact, the Alzheimer’s Drug Discovery Foundation has provided funding to manufacture clinical-grade senicapoc and conduct the required stability testing in support of an investigational new drug (IND) filing. Upon approval of the IND filing, senicapoc will be tested in a phase II clinical trial in prodromal and mild AD (https://www.alzdiscovery.org/news-room/announcements/1.8-million-in-new-funding-supports-clinical-stage-treatments).

Senicapoc has been through extensive preclinical safety testing as well as phase I, II, and III clinical trials with few significant side effects noted. Given our knowledge of the pharmacokinetics of senicapoc, including its CNS penetrance (Staal et al., 2017b), it is likely that a dose similar to that used in the previous clinical trials will be used. AD patients, however, are very different from patients with sickle cell disease. It is likely that the AD patients will be older and have more comorbidities than those with sickle cell anemia. The age and comorbidities could reveal underlying susceptibilities to K_Ca_3.1 inhibition that may not have been problematic in younger patients with sickle cell disease (e.g., susceptibility to infections). On the other hand, many diseases or conditions afflicting the elderly have inflammatory components, and a mild immunosuppressant such as senicapoc may benefit those conditions as well (e.g., stroke, chronic and neuropathic pain, atherosclerosis).

Yet, the current AD study is still incredibly important to test the hypothesis that targeting neuroinflammation, specifically K_Ca_3.1, can slow or halt the progression of AD. If the trial results demonstrated significant and convincing slowing of disease progression, pharmaceutical companies could initiate drug discovery projects to develop improved CNS penetrant K_Ca_3.1 inhibitors.

### Targeting the Kynurenine Pathway to Treat AD

Development of drugs that target products of tryptophan metabolism has transformed the treatment of psychiatric disorders, particularly with the advent of serotonin (5-HT) reuptake inhibitors (Wong et al., 1995). However, it is noteworthy that metabolism to 5-HT represents only a small fraction of the fate of free tryptophan, and in fact, the great majority is converted to KYN (Leklem, 1971) and its subsequent products by an array of enzymes located throughout the body and brain. Yet, surprisingly, there are no marketed medications that directly target metabolism of the KYN pathway.

**Figure 3:**
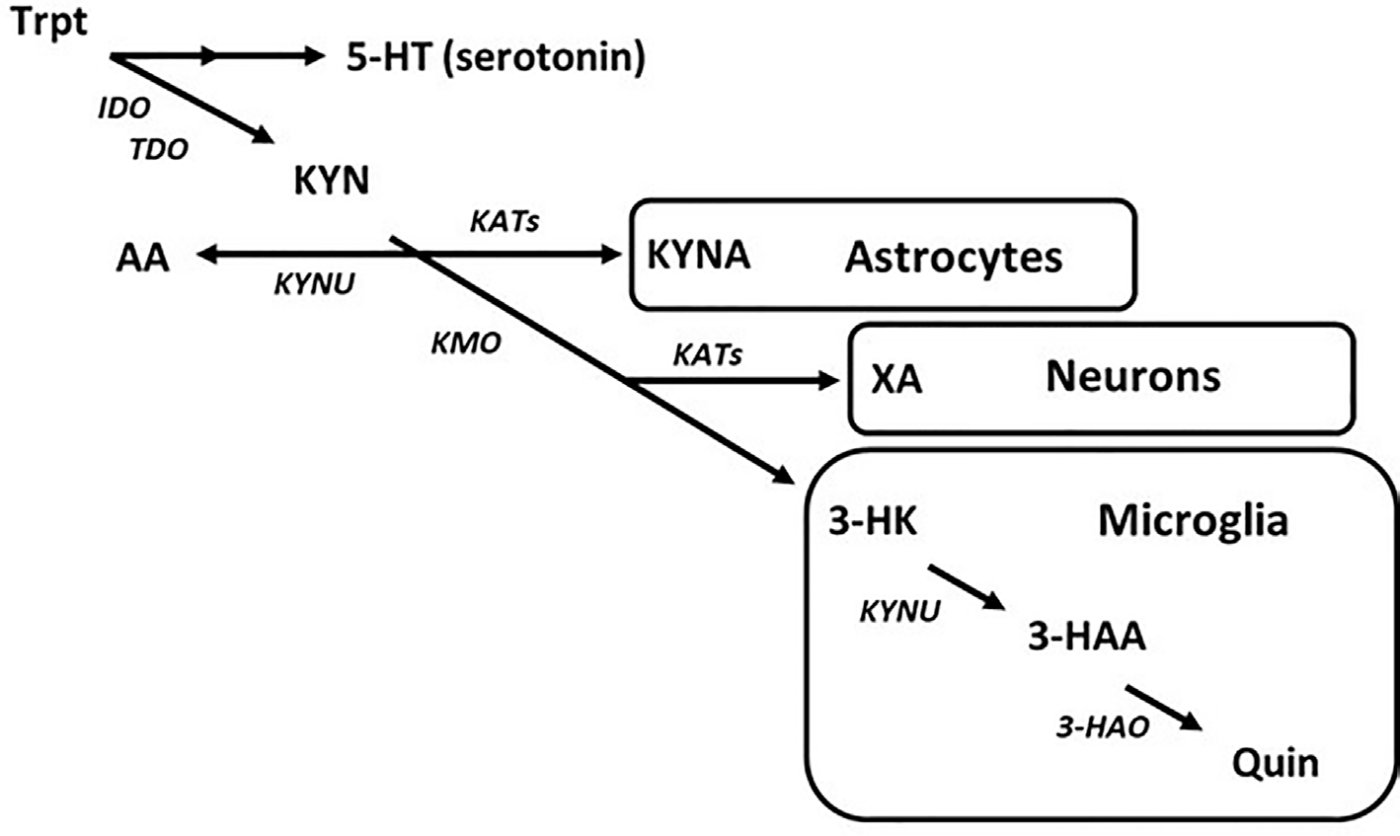
The kynurenine pathway of tryptophan metabolism in the brain. Simplified diagram depicting the tryptophan–kynurenine pathway. Metabolism of tryptophan along the kynurenine pathway in the brain is regulated by a variety of enzymes that are largely segregated by cell type.

Entry into the KYN pathway begins with metabolism of tryptophan into N-formylkynurenine by indole-2,3-dioxygenase (IDO) or tryptophan-2,3-dioxygenase (TDO), which is then rapidly converted to L-kynurenine (Takikawa et al., 1986; Zhang et al., 2007). Metabolism of KYN produces a host of neuro-active products in the brain that may contribute to CNS diseases (Török et al., 2016; Lovelace et al., 2017; Schwarcz and Stone, 2017). The KYN pathway bifurcates into neurotoxic and neuroprotective metabolic routes. As an example, astrocytes largely express kynurenine aminotransferases (KATs) and so are the primary source of the neuroprotective metabolite, kynurenic acid (KYNA), in the brain (Guidetti et al., 2007). Alternatively, microglia express kynurenine 3-monooxygenase (KMO), which converts KYN to neurotoxic metabolites such as 3-hyroxykynurenine (3-HK) and subsequently quinolinic acid (Quin) (Heyes et al., 1996; Guillemin et al., 2003a) (**Figure 3**). While the topic of this discussion is targeting the disruption in microglial KYN metabolism, one should understand that the various branches of KYN metabolism do not act in isolation, and so a change in one may cause a change in the others by altering the availability of substrate.

Inflammation triggers activation of microglia and dysregulation of KYN metabolism, leading to production of neurotoxic metabolites from the KMO branch, a process that is predicted to be involved in the development of AD pathology (Campbell et al., 2014; Zádori et al., 2018). However, direct evidence of microglial KYN metabolism from human AD subjects suggests that this effect may be limited to localized pathological events rather than global changes in CNS KYN metabolism. As an example, levels of KYN and 3-HK were both surprisingly reduced in CSF of AD patients with dementia (Tohgi et al., 1992). However, tryptophan, 5-HT, and melatonin were all also significantly reduced in the same study. In addition, the same group reported that KYN was reduced in a second cohort of patients with AD dementia but not in ones with vascular dementia (Tohgi et al., 1995). Furthermore, Quin (Mourdian et al., 1989; Heyes et al., 1992) and KYNA (Wennström et al., 2014) were reportedly unchanged in recent studies, though earlier evidence indicated that KYNA could also be decreased (Heyes et al., 1992; Hartai et al., 2007; Gulaj et al., 2010) in AD patients. Overall, there is limited evidence that CSF tryptophan/KYN metabolites are reproducibly altered in AD, though where it has been assessed, most studies point to either no consistent changes in metabolites or a reduction in KYN metabolism (**Table 1**).

**Table 1:** Kynurenine metabolite profile from patients with Alzheimer’s Disease.

Biosample	Metabolite	Effect	Reference
Serum	Trpt	Decreased No Change	Widner et al., 1999; Widner et al., 2000 Schwarz et al., 2013; Oxenkrug et al., 2017
	KYN	Increased No Change	Widner et al., 1999; Widner et al., 2000; Atukeren et al., 2018 Oxenkrug et al., 2017
	AA	Decreased	Oxenkrug et al., 2017
	XA	ND	
	KYNA	No Change	Schwarz et al., 2013; Oxenkrug et al., 2017
	3-HK	Increased	Schwarz et al., 2013; Oxenkrug et al., 2017
	Quin	No Change	Schwarz et al., 2013
Plasma	Trpt	Decreased	Greilberger et al., 2010; Giil et al., 2017
	KYN	No Change	Greilberger et al., 2010
	AA	Increased	Chouraki et al., 2017
	XA	Decreased	Giil et al., 2017
	KYNA	Decreased	Heyes et al., 1992; Hartai et al., 2007; Gulaj et al., 2010
	3-HK	Increased	Gulaj et al., 2010
	Quin	Increased Decreased	Gulaj et al., 2010 Giil et al., 2017
CSF	Trpt	Decreased	Tohgi et al., 1992; Muguruma et al., 2018
	KYN	Decreased	Tohgi et al., 1992; Tohgi et al., 1995; Muguruma et al., 2018
	AA	Decreased	Muguruma et al., 2018
	XA	ND	
	KYNA	No Change Decreased	Wennström et al., 2014 Heyes et al., 1992; Hartai et al., 2007; Gulaj et al., 2010
	3-HK	Decreased	Tohgi et al., 1992; Muguruma et al., 2018
	Quin	No Change	Mourdian et al., 1989; Heyes et al., 1992
Brain (post mortem)	KYN	No Change (regional)	Baran et al., 1999
	AA	ND	
	XA	ND	
	KYNA	No Change Increase (caudate/putamen)	Beal et al., 1992 Baran et al., 1999
	3-HK	No Change (regional)	Baran et al., 1999
	Quin	No Change (regional) Increased (plaques/tangles)	Moroni et al., 1986; Mourdian et al., 1989; Sofic et al., 1989; Pearson and Reynolds, 1992; Guillemin et al., 2005; Wu et al., 2013

In post-mortem AD brain tissue, early studies reported a lack of change in KYN metabolism. Indeed, Quin levels were shown to be unchanged (Moroni et al., 1986; Mourdian et al., 1989; Sofic et al., 1989; Pearson and Reynolds, 1992), as were KYNA concentrations (Flint Beal et al., 1992). However, more recent analysis revealed regional changes that indicated greater production of KYN as well as shunting of KYN metabolism toward production of neurotoxic microglial metabolites. Immunohistochemical analysis showed that both TDO and IDO were elevated in the hippocampus of AD patients (Guillemin et al., 2005; Wu et al., 2013). Importantly, the increase in IDO expression was found in microglia, astrocytes, and neurons, with the primary product found in proximity to senile plaques and neurofibrillary tangles in post-mortem AD brain being Quin, the microglial product (Guillemin et al., 2005; Bonda et al., 2010). These data are in line with reports that treatment of microglia with Aβ_1–42_ induces IDO expression (Guillemin et al., 2003a; Guillemin et al., 2003b) and that Quin may seed α-synuclein aggregation (Tavassoly et al., 2018) and induce tau hyperphosphorylation (Rahman et al., 2009). Given the well-documented evidence that tryptophan and KYN metabolism is sensitive to inflammatory stimuli (Campbell et al., 2014; Strasser et al., 2017), it is plausible to predict that neuroinflammation centered around plaques and tangles in the brains of AD patients produces localized pro-inflammatory activation of microglia, resulting in dysregulation of KYN metabolism, which favors production of neurotoxic metabolites such as 3-HK and Quin. A deleterious impact resulting in local brain circuit disruption and longer-term neurodegeneration is hypothesized, but further analysis in longitudinal studies is needed to confirm or disprove this.

#### Challenges of Developing Kynurenine Pathway Drugs for AD

The main challenge for developing KYN metabolite inhibitors for AD is a clear understanding of the role of KYN metabolic products in the development and/or progression of AD, as well as the availability of agents to test the efficacy of CNS inhibition of neurotoxic KYN enzymes, specifically KMO. Peripheral measures of KYN metabolism in serum and plasma support the hypothesis that the KYN pathway is induced in AD, leading to increased production of KYN and products associated with the microglial metabolic branch. However, it is unclear whether this peripheral pattern of metabolism reflects CNS KYN metabolism or contributes to neural pathology. Indeed, distribution of metabolites in the CNS (brain, CSF) suggest a more nuanced pattern of regulation that also supports a shunting of tryptophan metabolism toward neurotoxic KYN metabolites, but only in proximity to plaques and tangles, indicating a need for brain-penetrable KMO inhibitors to act locally at the site of pathology. As discussed above, there is also a lack of longitudinal data on KYN metabolism in AD patients and an analysis of how these data correlate with disease progression or severity state. The limited correlation between blood and CSF metabolite profiles, as well as with post-mortem brain patterns, highlights the need for methodologies that correlate disease progression with direct measurements of brain KYN metabolites and/or enzymes in living AD patients. A concerted effort to develop positron-emission tomography (PET) ligands and/or other imaging agents targeting the KYN pathway would be a great benefit toward understanding the role of this pathway in AD and improving the rationale for the substantial investment required to develop brain-penetrant drugs.

While KMO has been targeted by the pharmaceutical industry for at least 3 decades, there are no published agents with good CNS drug exposure. Importantly, targeting KMO in the periphery creates a substantial increase in substrate, KYN, which is actively transported into the brain, where it is converted to substances such as KYNA, which could be neuroprotective, but also into 3-HK and Quin (Beaumont et al., 2016), which may increase oxidative stress and neuronal damage. This could be especially problematic in neurodegenerative disorders such as AD where neuroinflammation associated with plaques and tangles is predicted to increase KMO expression, thus more readily converting KYN to 3-HK and Quin (Guillemin et al., 2003a; Guillemin et al., 2005; Bonda et al., 2010). Therefore, it will be necessary to focus drug discovery efforts on identifying KMO inhibitor chemotypes with a high degree of CNS penetrance to test their therapeutic benefit in AD. Indeed, it may even be advantageous to attempt developing non-competitive KMO inhibitors along the lines of recent advances in KAT II inhibitors (Dounay et al., 2012) since they would not compete with increased KYN pumped into the brain from the periphery that could otherwise limit their efficacy. Finally, while the paucity of brain-penetrant drugs targeting the KMO branch of the KYN pathway is discouraging, advances have been made in developing agents that stimulate the neuroprotective KAT branch, such as 4-Chlorokynurenine (4CL-KYN; AV-101). Peripheral administration of 4Cl-KYN is transported into the brain, where it is converted to 7-chloro-KYNA, thereby acting as a locally synthesized KYNA mimetic (Hokari et al., 1996), where it appears to have promising neuroprotective effects (Wu et al., 2000; Wu et al., 2002). These data are encouraging and support the hypothesis that development of centrally acting KMO inhibitors could be neuroprotective in AD since blocking microglial KYN metabolism would not only prevent production of neurotoxic metabolites such as 3-HK and Quin but also indirectly increase endogenously produced KYNA. Thus, a greater understanding of the impact of rebalancing the neurotoxic and neuroprotective branches of the KYN pathway in AD may provide additional mechanistic evidence to support development of a new generation of neuroprotective brain-penetrant KMO inhibitors.

### The P2X7–NLRP3 Axis in Alzheimer’s Disease

The P2X7–NLRP3 (nucleotide-binding oligomerization domain, leucine-rich repeat, and pyrin domain–containing inflammasome complex 3) pathway has received extensive attention, with several publications pointing to its potential role in neuroinflammatory disorders, from psychiatry to neurodegenerative diseases. Microglia express several cell-surface and cytoplasmic sensors of danger signals that react to invading “dangers” and cause neuroinflammation. The ATP-gated ion channel P2X7 is expressed abundantly in microglia and responds to extracellular ATP, which often occurs during tissue distress or damage and can lead to activation of the NLRP3 inflammasome complex, producing pro-inflammatory cytokines, IL-1β and IL-18 (Bhattacharya and Biber, 2016). As such, the P2X7–NLRP3 pathway has been the focus of intense drug discovery efforts: while brain-penetrant P2X7 antagonists have made progress and are in the clinic (**Table 2**), brain-penetrant, drug-like NLRP3 inhibitors are the focus of medicinal chemistry discovery programs.

**Table 2:** Clinical strategies used to dampen IL-1β signaling.

P2X7 Antagonists	Indication(s)	Comments
CE-224,535 (Pfizer)	Rheumatoid arthritis Not in development	Failed efficacy study (Ph-II) Not brain penetrant Blocked ex-vivo human IL-1β
AZD-9056 (AstraZeneca)	Rheumatoid arthritis Crohn’s Not in development	+ve signal in Chron’s +ve & -ve signals in RA Blocked ex vivo human IL-1β Not brain penetrant
GSK-1482160 (GlaxoSmithKline)	Pain (intended) Not in development	Phase-I safety study Blocked ex-vivo human IL-1β Brain penetrant
SGM-1019 (Second Genome)	NASH (intended) In active development	Phase-I safety study No information on compound
JNJ-54175446 (Janssen)	CNS In active development	Phase-I safety study Blocked ex-vivo human IL-1β Brain penetrant
JNJ-55308942 (Janssen)	CNS In active development	Phase-I safety study Brain penetrant
NLRP3 Inhibitors	Indication(s)	Comments
OLT-1177 (Olatec)	Inflammatory disorders In active development	Phase-II (CAPS) No ex-vivo human IL-1β data In-vitro blockade of IL-1β No brain penetration data
Caspase-1 inhibitors	Indications	Comments
VX-765 (Vertex)	Epilepsy Psoriasis Not in development	Brain penetrant
Vx-740 (Vertex)	Rheumatoid arthritis Psoriasis Not in development	Pro-drug
IL-1β biologics	Indications	Comments
Anakinra	Rheumatoid arthritis	IL-1r antibody Marketed product (injection)
Rilonacept	Inflammatory disorders CAPS, FCAS MWS	IL-1β and IL-1α antibody Marketed product (injection)
Canakinumab (Novartis)	Atherosclerosis Lung cancer	IL-1β antibody Phase-III

P2X7 belongs to the P2X family of trimeric ligand–gated cation channels. Its activation by ATP allows for the influx of several cations, including Ca^2+^, Na^+^, and K^+^ (Bartlett et al., 2014). Even though P2X7 expression is abundant in microglia (and in peripheral immune cells), it is “silent” under normal physiology, where ATP concentrations do not reach the high micromolar levels required to activate its ion channel (Bhattacharya and Biber, 2016). As such, P2X7 is an attractive drug target as antagonism of a silent channel by true neutral antagonists would not cause any serious target mediated (adverse) effects: antagonism will only be evident when the channel is activated by high ATP concentrations, which is believed to occur during neuroinflammatory disorders of the CNS. NLRP3, on the other hand, is a convergent point of inflammasome activation, both dependent and independent of P2X7 activation (Bhattacharya and Jones, 2018), and as such is an attractive drug target. The advantages and disadvantages of targeting P2X7 vs. NLRP3 in neuroinflammatory disorders remain to be determined.

The P2X7–NLRP3 pathway is a case in point where microglia-driven neuroinflammation may play a role in AD causality (Heneka et al., 2013), and there has been growing interest in probing the role of pathogenic Aβ in neuroinflammation, although the exact mechanism of its involvement is still unknown (Illes et al., 2019; Martin et al., 2019; Thawkar and Kaur, 2019). P2X7 activation and consequent release of IL-1β is one such mechanism of interest in the AD field in the quest to understand the role of neuroinflammation in AD (Sanz et al., 2009). P2X7 upregulation has been reported in post-mortem AD brains and in animal models of tauopathy and amyloid deposition, mostly around the areas of pathology (Parvathenani et al., 2003; McLarnon et al., 2006; Lopez-Gonzalez et al., 2015). For example, P2X7 upregulation was seen in cortical samples from AD patients compared to healthy volunteers (Martin et al., 2019). With the recent discoveries of clinically available P2X7 PET ligands (Bhattacharya, 2018), the detection of P2X7 upregulation in humans becomes possible. In a mouse model of AD/frontotemporal lobar degeneration (FTLD) (P301S), enhanced uptake of P2X7 PET signal was detected in brain regions of tauopathy (Jin et al., 2018). P2X7 activation has also been linked to neuronal damage and synaptotoxicity in transgenic AD models (Lee et al., 2011). There are signals of an active P2X7–NLRP3 axis in animal models of AD. For example, in the J20 mouse model of AD, which overexpresses the human APP gene with two familial AD-linked mutations, the authors noted that P2X7 activation prevents APP processing by α-secretase, facilitating the formation of toxic Aβ *via* P2X7-mediated activation of glycogen synthase kinase 3 (GSK-3) (Diaz-Hernandez et al., 2012). Supporting these data, inhibition of P2X7 with Brilliant Blue G (BBG) in the same mouse model increased α-secretase activity and reduced the formation of Aβ plaques, supporting a beneficial role of P2X7 antagonism in AD (Chen et al., 2014). Recently, it was reported that mice lacking P2X7 have reduced Aβ load and cognitive impairment in another AD model overexpressing both human mutated APP and mutated presenilin-1 (APP–PSEN1) (Martin et al., 2018). In addition to P2X7, the downstream intracellular NLRP3 inflammasome has been receiving special attention through several publications, and there is a growing interest to bring forward NLRP3 inhibitors for clinical testing in AD (Heneka, 2017; Heneka et al., 2018). For example, MCC950 (NLRP3 inhibitor) has been reported to prevent cognitive decline in APP/PS1 mice (Dempsey et al., 2017). A comprehensive study by Venegas et al. demonstrated mechanistically that ASC [adapter protein apoptosis-associated speck-like protein containing a caspase activation and recruitment domain (CARD)] specks (involved in NLRP3 activation) contributed to the spreading of Aβ plaques in APP/PSEN1 double-mutant mice (Venegas et al., 2017). The study by Martin et al. points to the unique role of P2X7, outside the NLRP3–IL-1β signaling cascade. While support for the NLRP3 pathway being involved in disease is gaining momentum, there are subtleties of targeting P2X7 vs. NLRP3 inflammasome with therapeutics. P2X7 antagonism may bring additional benefits as highlighted in the study by Martin et al. (2018). While NLRP3 is downstream of P2X7 and may be a critical mediator of IL-1β release through the inflammasome, P2X7 antagonism may provide additional benefits such as modulation of synaptic plasticity, modulation of chemokine release (e.g., CCL3), and consequent effects on CD8^+^ T cell recruitment to focal regions of the diseased brain.

#### Challenges Around P2X7/NLRP3

While the scientific community continues to generate data supporting the role of P2X7–NLRP3 in models of AD, there must be a healthy balance of caution and opportunistic investments in clinical trials to test these new mechanisms. The biggest challenge of NLRP3 for AD is a lack thus far of a suitable clinical compound with acceptable brain penetration. For P2X7, several brain-penetrant antagonists have been disclosed, some of which are in clinical development (**Table 2**). With the availability of brain-penetrant P2X7 antagonists and P2X7 PET ligands, these clinical tools could be tested in a patient population, perhaps with the help of PET and associated biomarkers (CSF, blood, genomics) that would enrich AD patients with an active P2X7–NLRP3 axis. In addition to using P2X7 PET ligands as imaging tools to enrich patient clusters for proof-of-concept testing, one must also demonstrate target engagement at clinically achievable doses. Target engagement in patients receiving a clinical candidate for proof-of-concept testing is critical to the evaluation of primary end points. Emerging data supporting the role of microglia-driven processes (neuroinflammation, phagocytosis), and description of different phenotypes of microglia in normal and disease states has prompted renewed thinking regarding their role in AD. To that end, it is of some comfort to witness that the role of microglia and microglia-driven neuroinflammation in AD has been the subject of some high-profile publications in the last few years, including significant progress with clinical compounds. While brain-penetrant P2X7 compounds are clinically available (**Table 2**), NLRP3 inhibitors that adequately demonstrate central target engagement (with NLRP3 PET ligands) are currently lacking. Demonstration of central target engagement is fundamental to drug development; once a dose-occupancy is established, moving to AD patients is the next step, with the challenge of selecting patients who can be intervened in early on in the disease process.

### Proposed Role of PD-1 Immune Checkpoint Blockade in AD

PD-1 (or CD279) is a member of the CD28 family of T cell regulators (Riley and June, 2005). It is weakly expressed on naive T cells but can be induced upon activation in several types of immune cells, including B cells, T cells, natural killer cells, dendritic cells, and monocytes. PD-1 plays an important role in immune responses related to B cell activity and/or T cell activity, such as antibody production, immune tolerance, and autoimmunity (Okazaki et al., 2013). PD-1 function is important to preserve immune tolerance. Taken together, PD-1 signaling ameliorates immune overreaction.

Recent studies have indicated that boosting the immune response in the brain might have therapeutic potential. It was shown that inhibition of forkhead box protein P3 (FOXP3)-positive regulatory T cells increased interferon-γ (IFNγ)–dependent leukocyte trafficking to the brain, promoting clearance of Aβ and improving cognitive function in a mouse model of amyloidosis (Baruch et al., 2015). As PD-1 checkpoint blockade was previously reported to stimulate IFNγ-dependent immune responses in cancer immunotherapy, the therapeutic potential of PD-1 blockade in AD models was explored (Baruch et al., 2016).

In their studies, the authors treated the 5XFAD mouse model of amyloidosis at 10 months of age (an age with advanced pathology) with two intraperitoneal injections of a PD-1–specific antibody with 3-day intervals. One week after the first treatment, the mice were reported to exhibit a systemic IFNγ immune response. Further analysis of the myeloid cell populations in the brain upon treatment documented IFNγ-dependent recruitment of monocyte-derived macrophages as characterized by high expression of lymphocyte antigen 6c and expression of the chemokine receptor CCR2. Intriguingly, treatment also resulted in reduced cognitive deficits. In detail, spatial learning and memory in the radial-arm water-maze task was improved 1 month after treatment. Furthermore, 2 months after initial treatment, 5XFAD mice that had received 2 rounds of the anti–PD-1 treatment at a 1-month interval performed indistinguishably from non-transgenic mice. In comparison, mice with a single round of PD-1 blockade that were examined 2 months later were not different from placebo control and hardly showed improved memory. These results indicated that repeated treatment was needed to maintain improved cognition. Treatment with the PD-1–specific antibodies also caused a reduction in plaque load in the hippocampus and in the cortex. This treatment effect was more pronounced after two rounds of anti–PD-1 treatment. In addition, astrogliosis was reduced in the hippocampus of 5XFAD mice with either one or two rounds of anti–PD-1 treatment. The beneficial effects described in 5xFAD mice after PD-1 blockade were largely confirmed in a second mouse model of amyloidosis (APP/PS1APPswe, PSEN1dE9), in which treatment reduced plaque deposition in hippocampus.

Given the fact that various antibodies or small molecules targeting PD-1/PD-1L are already marketed or under development (Kobold et al., 2018; Shaabani et al., 2018), the findings by Baruch et al. triggered considerable interest in immune checkpoint blockade as a novel therapeutic strategy for AD. Accordingly, this study stimulated evaluation of the PD-1 immune checkpoint blockade for AD by three independent pharmaceutical companies.

#### Challenges of PD-1 Immune Checkpoint Blockade as Therapeutic Intervention in AD

In subsequent studies jointly published in *Glia* (Latta-Mahieu et al., 2018) by Sanofi, Janssen, and Eli Lilly, the consequences of PD-1 immunotherapy in a range of different APP transgenic models (ThyAPP/PS1M146L, ThyAPP/PS1A246E, PD-APPAPPV717F) with the same anti–PD-1 antibody were documented. In addition to the antibody used by Baruch et al., two mouse chimeric variants and corresponding Immunglobulin G (IgG) controls were tested. In accordance with Baruch et al. (2016), PD-1 immunotherapy caused a systemic IFNγ-dependent immune response (Latta-Mahieu et al., 2018). However, in contrast to Baruch et al. (2016) there was no effect of anti–PD-1 treatment either on amyloid pathology or on monocyte-derived macrophage infiltration into the brain. This result point is consistent with data from another laboratory that explored the consequences of PD-1 deficiency in a murine prion disease model of neurodegeneration (ME7). In this study, there was no detectable infiltration of peripheral myeloid cells into the brain (Obst et al., 2018).

Latta-Mahieu and coworkers used, in addition to the rat mouse antibody (mAb) used by Baruch et al., two mouse chimeric variants with mouse IgG1 and IgG2a Fc domains as additional controls to evaluate whether observed effects were directly related to PD-1 target engagement. The *in vivo* studies were designed to ensure high statistical power (n = 9/group at Sanofi, n = 20/group at Janssen, and n = 25/group at Eli Lilly) and carried out in a blinded fashion. Furthermore, additional cohorts of animals for baseline measurement of pathology at the start of the anti–PD-1 treatment have been included. This way, age-dependent increases in amyloid pathology in each model of amyloidosis could be compared to anti–PD-1 treatment effects at the end of the study (Latta-Mahieu et al., 2018).

The reason for the different findings between Baruch et al. and Latta-Mathieu et al. is unclear, and more research is needed to understand the potential of PD-1 as a target for AD. It should be noted that in the study by Baruch et al., a xenogeneic rat antibody was used that potentially made the interpretation of the results difficult. Thus, it remained uncertain whether PD-1 target engagement or stimulation of the immune system, independent of PD-1, was causing the reduction in pathology and the functional improvements.

### The Proposed Role of Toll-Like Receptors in AD

The innate immune system is the first line of defense against pathogens and tissue damage and elicits a rapid and robust response. Within the CNS, the innate immune system performs a similar role that is mediated by microglial and astroglial cells (Ransohoff and Brown, 2012). In the case of CNS infection or injury, microglial cells detect pathogen-associated molecular patterns (PAMPs) or danger-associated molecular patterns (DAMPs) *via* specialized cell-surface pattern recognition receptors (PRRs). PRRs transmit information about the CNS microenvironment to the glial cells, which then orchestrate an appropriate immune response (Kigerl et al., 2014). Glia direct the neuroimmune response by migrating toward lesions or pathology, producing pro- and anti-inflammatory cytokines and chemokines, releasing growth factors and up regulating phagocytic mechanisms (Hansen et al., 2018). After a period, there is a resolution signal, which returns glia to their homeostatic state (Chavan et al., 2017). However, in AD, where Aβ, hyperphosphorylated tau, and neuronal damage persist for decades, there is no resolution, resulting in chronic activation of the innate immune system. This persistent alteration of glial cell phenotype is believed to contribute to AD progression and severity, and therefore, the identification of drugable molecular targets within the innate immune system of the CNS is of paramount interest.

TLRs are a major class of PRRs characterized by an extracellular leucine-rich repeat domain and an intracellular TIR domain (Kielian, 2006). Ten mammalian TLRs have been identified, each with a particular ligand specificity. Complicating the picture, TLRs can hetero-dimerize with additional co-receptors, resulting in an expansion of their ligand repertoire (Kielian, 2006). TLRs are expressed in microglia (Laflamme and Rivest, 2001; Laflamme et al., 2001; Bsibsi et al., 2002; Dalpke et al., 2002; Olson and Miller, 2004; Zhang et al., 2012), and several have been shown to have increased expression both in the brains of AD transgenic animals and in human AD patients (Liu et al., 2005; Walter et al., 2007; Frank et al., 2009; Letiembre et al., 2009; Wirths et al., 2010; Rangasamy et al., 2018). The primary consensus among researchers is that persistent TLR signaling in AD models is detrimental; however, conflicting evidence also suggests beneficial roles of TLR signaling. The most extensively studied TLRs, in the context of AD, are TLR2 and TLR4 along with their co-receptor, CD14.

TLR2 and its co-receptor CD14 are upregulated in AD transgenic mouse models and in human AD brain sections (Letiembre et al., 2009). Functional characterization of TLR2 in primary mouse microglia and the mouse BV-2 microglia cell line demonstrated that fibrillar preparations of Aβ peptide can increase expression of NO synthase, pro-inflammatory cytokines, and integrin markers in a TLR2-dependent manner (Jana et al., 2008). Consistent with this, primary microglia and bone marrow–derived macrophages from TLR2 knockout mice show a reduced pro-inflammatory reaction and an enhanced phagocytic response when stimulated with aggregated Aβ_1–42_ peptide (Liu et al., 2012). Anti-TLR2 antibodies, administered over seven months, improved spatial learning, reduced gliosis [as assessed by immunoreactivity of CD68 and glial fibrillary acidic protein (GFAP)], and decreased Aβ plaque burden (McDonald et al., 2016). Anti-TLR2 antibodies can prevent a metabolic switch in microglia toward glycolysis, which results in an increase in phagocytosis (Rubio-Araiz et al., 2018). A peptide corresponding to the TLR2-interacting domain of myeloid differentiation primary response 88 (MyD88) (TIDM) disrupted the interaction of TLR2 with MyD88, resulting in a blockade of TLR2 signaling (Rangasamy et al., 2018). When administered intranasally to 5xFAD transgenic mice, the peptide could be detected in the hippocampus, where it reduced glial activation and Aβ plaque burden (Rangasamy et al., 2018). In contrast, several studies have postulated that TLR2 can promote beneficial responses in AD models. TLR2 deficiency accelerated spatial and contextual memory deficits while increasing levels of Aβ_1–42_ in the brains of PS1/APP transgenic mice (Richard et al., 2008). In addition, the TLR2 agonist peptidoglycan (PGN) enhanced uptake of Aβ_1–42_ in primary mouse microglia (Chen et al., 2006).

Like TLR2, TLR4 is expressed in microglial cells and can mediate binding and internalization of Aβ_1–42_ *in vitro* (Tahara et al., 2006). TLR4 was originally identified as an endotoxin receptor, mediating immune response to stimuli such as LPSs (Janssens and Beyaert, 2003). Studies using LPS as a tool to activate TLR4 signaling in AD transgenic mice have produced conflicting results. Intra-hippocampal administration of LPS to Tg2576 or PS1/APP mice activated microglia (as assessed by CD45 and CR3 or MHCII immunoreactivity) and reduced diffuse Aβ staining but not compact plaque (DiCarlo et al., 2001; Herber et al., 2007). Multiple doses of peripheral LPS improved cognitive function and reduced tau pathology in the P301S model (Qin et al., 2016). In contrast, repeated peripheral administration of LPS to WT mice was shown to increase Aβ levels, activate microglia, and induce cognitive impairment (Lee et al., 2008; Lee et al., 2012). Studies with in APPswe PS1dE9 and PS1/APP TLR4 knockout mice confirm an increase in plaque pathology (Tahara et al., 2006; Song et al., 2011), resulting in reduced glial activation and improved cognitive function (Song et al., 2011). Therapeutic blockade of TLR4 signaling using the cyanobacterial product CyP (Macagno et al., 2006) injected intracerebroventricularly (ICV) was able to reduce memory impairment and glial cell activation induced by ICV administration of Aβ oligomers (Balducci et al., 2017).

#### Challenges Targeting TLRs

Over the last two decades, considerable attention has been paid to understanding the role of TLRs in innate immunity, but their role in AD initiation and progression remains elusive. As highlighted above, the transgenic models used, the timing of the intervention, and the end points measured can impact the conclusions made. AD is a complex disease, and the effects of modulating TLRs may differ depending on the stage of the disease during intervention. For instance, memory impairment was reduced after ICV injection of Aβ oligomers by pre-treatment with TLR2 and TLR4 agonists (Pourbadie et al., 2018). In contrast co-administration of TLR4 antagonist produced similar results (Balducci et al., 2017). Additional complexities in targeting TLRs arise from their ability to downregulate the immune response following repeated exposures to TLR agonists like LPS. This “endotoxin tolerance” has been described in animals and a variety of human diseases (Lopez-Collazo and del Fresno, 2013): this might confound interpretation of experimental data using TLR agonists. This is highlighted by recent work that demonstrated that the microglia response to LPS increases in aged WT animals compared to young WT mice. However, in the PS1/APP model, the microglia response to LPS is decreased in aged animals compared to WT (Go et al., 2016). This altered TLR signaling (i.e., tolerance) could affect the efficacy of TLR agonists or antagonists especially where chronic administration of the drug will be needed, such as in AD.

TLRs are expressed on a wide variety of cells, including peripheral immune cells and non-immune cells within the CNS such a neurons (Kielian, 2009). With traditional small molecule approaches, selectively modulating CNS and not peripheral TLRs is not feasible. Therefore, a suitable therapeutic window will need to be established for any TLR therapeutic developed. This will require a delicate balance between achieving the brain exposures needed to drive a therapeutically beneficial effect and minimizing peripheral exposures to reduce the risk for potential toxicities. The typically lower brain exposure compared to the periphery that is observed for many drugs adds to this challenge.

Several small molecule TLR agonists and antagonists have been described (Zhu et al., 2018). Discovery of TLR8 inhibitors was greatly facilitated by structure-based design (Hu et al., 2018). Similar approaches may be useful in designing small molecules for additional TLRs. Rather than drugging TLRs directly, others have sought to interfere with key protein–protein interactions required for TLR signaling. The TIDM peptide serves as a MyD88 decoy, essentially capping TLR2, preventing assembly of the myddosome and subsequent TLR2 signaling (Rangasamy et al., 2018). TAK-242 is a small molecule that interacts with cysteine-747 in TLR4, preventing interaction with TIR domain–containing adaptor protein (TIRAP) or TRAM resulting in blockade of TLR4 signaling (Matsunaga et al., 2011). Degrader approaches may also be useful to selectively shut down TLR signaling, but the use of such technology has not been demonstrated on TLRs. The therapeutic potential of TLRs in AD remains compelling, and the recent development of selective TLR ligands will support investigations of the role of TLRs in AD while helping to frame a robust therapeutic hypothesis.

### Proposed Role of TREM2 in AD

TREM2 is an immunoreceptor of partially understood function, expressed in myeloid cells in the periphery including dendritic cells, tissue macrophages and osteoclasts, and brain microglia (Colonna and Wang, 2016). TREM2 is a prominent AD risk gene (Pimenova et al., 2018) and is remarkable in the context of AD for at least two reasons: 1) its selective brain expression in microglia clearly links microglia specifically to neurodegenerative disease, and 2) there is the possibility of pharmacologic modulation of TREM2 to treat neurodegenerative disease.

In 2013, two landmark papers (Guerreiro et al., 2013; Jonsson et al., 2013) identified, by exome sequencing, rare mutants in TREM2 associated with an AD risk similar to that of a single allele of the strongest known risk factor, the E4 isoform of Apolipoprotein E (ApoE). Specifically, they showed that the mutation R47H, present in about 0.2% of the human population, elevated the AD odds ratio to at least 3.0. Several subsequent studies found slightly lower odds ratios but have confirmed the TREM2 R47H observations and identified additional TREM2 risk alleles and variants of unknown consequence (Bellenguez et al., 2017; Sims et al., 2017) that, in total, are present in about 1.5% of the human population (http://exac.broadinstitute.org/). Many other genes of modest AD risk have been identified before and since (Pimenova et al., 2018). But TREM2 is the strongest risk factor expressed in the brain only in microglia, unambiguously linking innate immune mechanisms in general, and microglial biology in particular, to AD. This observation has focused a great deal of attention on the role of microglia in AD and encouraged exploration of novel microglia-specific approaches to AD therapy (Hansen et al., 2018; Song and Colonna, 2018).

**Figure 4:**
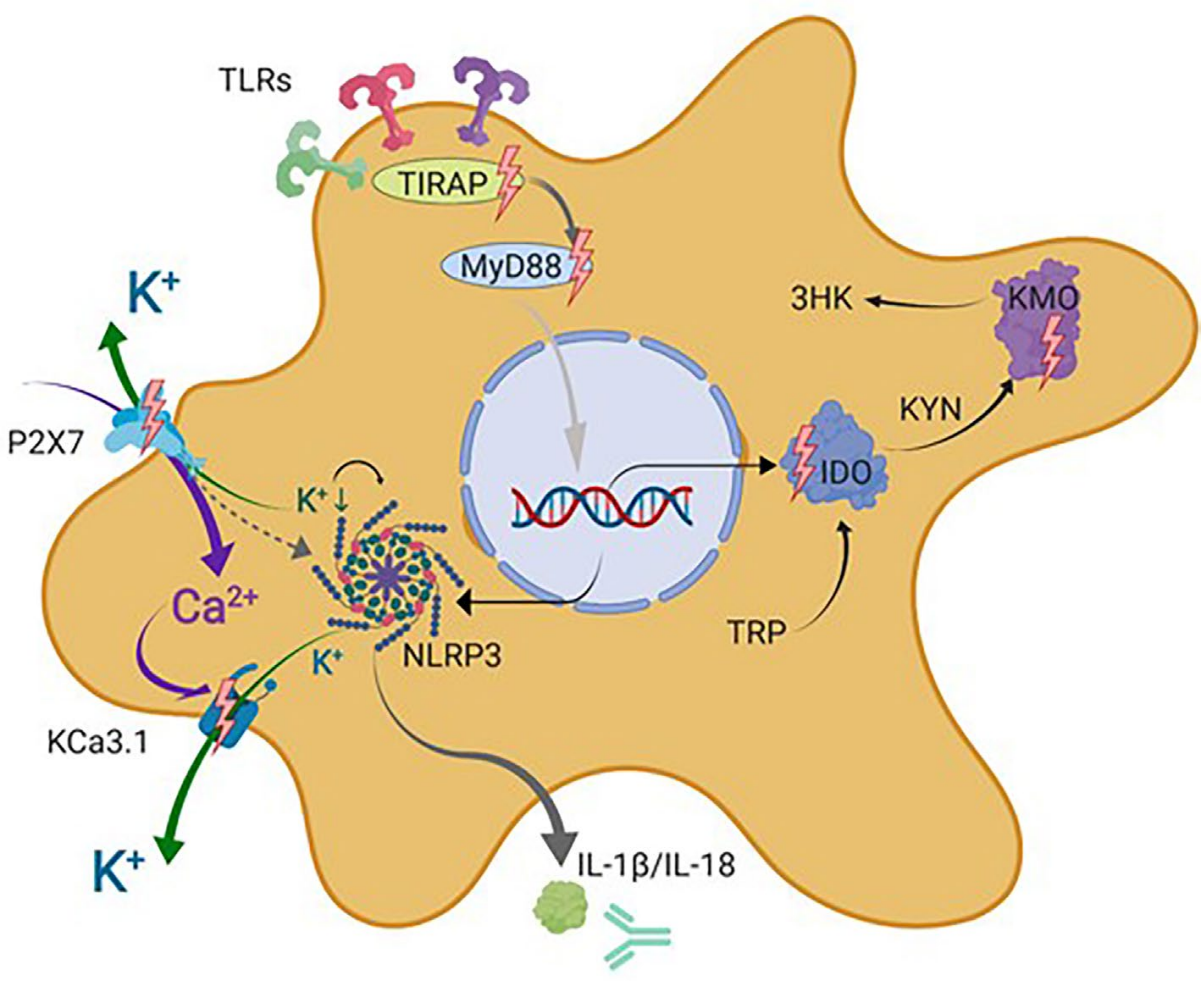
Schematic representation of microglial drug targets discussed in this review. This simplified schematic does not contain all signal transduction molecules known to be involved in the described signaling cascades but focuses on the microglial drug targets discussed in this review indicated by lighting bolts and antibody symbol. IDO, indolamine-2,3-dioxygenase; IL-1β, interleukin 1 beta; IL-18, interleukin 18; K_Ca_3.1, intermediate-conductance calcium-activated potassium channel 3.1; KYN, kynurenine; AA, anthranilic acid; 3-HK, 3-hydroxykynurenine; KATs, kynurenine aminotransferases; KMO, kynurenine 3-monooxygenase (kynurenine 3-hydroxylase); NLRP3, nucleotide-binding oligomerization domain, leucine-rich repeat, and pyrin domain–containing inflammasome complex 3; P2X7, ionotropic P2 receptor 7; TIRAP, TIR domain–containing adaptor protein; MyD88, myeloid differentiation primary response protein 88; TDO, tryptophan-2,3-dioxygenase; TRP, tryptophan. Figure created with Biorender.com

Functions of TREM2 in health and disease are summarized in several excellent reviews (for example, Hansen et al., 2018). AD-associated TREM2 mutants confer at least partially reduced ligand affinity, signaling, phagocytosis, and microgliosis (Ulrich et al., 2017). Significant evidence suggests that TREM2 is a direct phagocytic receptor (Takahashi et al., 2005; N’Diaye et al., 2009), which implies that failure to phagocytose aggregated proteins is a key driver of AD and related diseases. Note, however, that TREM2 is but one of many phagocytic receptors in microglia (Sierra et al., 2013), and it is unclear why the contribution of TREM2 in particular to phagocytosis would so strongly affect AD risk. Perhaps TREM2 has a prominent role in the phagocytosis of substrates specific to AD, e.g., Aβ or tau, or perhaps compromised TREM2 function has other pathological consequences (**Figure 4**). TREM2 also seems necessary for microglial plaque association in mouse models and in human disease (Wang et al., 2016; Yuan et al., 2016). Functional studies have suggested that TREM2 is a lipid receptor, and that the binding of some but not all lipids is compromised by AD-associated mutations (Wang et al., 2015). Evidence for other ligands including ApoE (Atagi et al., 2015; Bailey et al., 2015; Yeh et al., 2016) and Aβ peptide (Lessard et al., 2018; Zhao et al., 2018; Zhong et al., 2018) has been presented. These results should be interpreted cautiously. Aβ peptide is markedly hydrophobic, making specific interactions difficult to interpret. Also, depending on the presence and nature of bound lipids, ApoE is likely to have different interaction properties. The key ligand(s) for TREM2 in AD may not yet be known, or TREM2 may simply be a promiscuous receptor (Kober and Brett, 2017). The phenotype of mouse ApoE knockouts mimics TREM2 knockouts with respect to microglial plaque association (Shi and Holtzman, 2018; Ulrich et al., 2018), supporting ApoE as a TREM2 ligand and suggesting a model in which TREM2-mediated effects on plaque may require or be enhanced by a ternary complex with ApoE. Structural studies provide some insight into the specific effects of AD risk of TREM2 mutations. R47, for example, is a surface residue that may constitute part of the TREM2 ligand binding surface (Kober et al., 2016). In mutant form (R47H), histidine substantially alters the conformation of a surface loop, which may change the energetics of ligand binding (Sudom et al., 2018). It should be noted that some TREM2 mutants, including ones that eliminate expression by chain termination, are associated with a different neurologic condition called Nasu–Hakola disease (NHD). AD-associated mutations change amino acids on the outer surface of the protein and are predicted to have subtle structural effects. In contrast, NHD sequence variants are buried in the core of the protein and are predicted to have profound effects on protein structure and expression (Kober et al., 2016). Thus, relatively conservative mutations increase the risk of AD, while protein elimination is associated with NHD.

Some challenges in the TREM2 field have slowed progress in our understanding of its role in AD. Many studies are in cells other than microglia—often, peripheral macrophages. Many studies are done with TREM2 knockouts. As noted above, such knockouts are associated with NHD rather than AD, and the mechanistic relationships between the two are essentially not known. Some studies rely on overexpression of TREM2 (e.g., Jiang et al., 2016; Lee et al., 2018), which risks overwhelming TREM2 signaling partners in these cells, with uncertain consequences. Much of this is understandable. A major problem is that the phenotype of the AD risk sequence variants is quite subtle, making differential readouts and clear conclusions difficult. Some investigators use heterozygous as well as homozygous knockouts to reveal trends that are complex to interpret. Likewise, the relationship between a heterozygous knockout and, for example, the R47H sequence variant is not clear, and the latter is the preferable experimental target (for example, Cheng et al., 2018; Song et al., 2018). Compounding the TREM2 phenotype problem are difficulties common to all studies of microglia; these cells are extremely sensitive to their environment, rapidly changing their gene expression profile *ex vivo* (Bohlen et al., 2017). Human primary cells are available, of course, only from cadavers, and even then, only after several hours of hypoxia, with uncertain impact. Inducible pluripotent stem cell (iPSC)-derived “microglia-like” cells have, at best, an uncertain relationship with human microglia in their natural setting. Finally, due largely to the lack of reagents, intervention studies with pharmacologic modulators are rare: one such study using an antibody reported to activate TREM2 signaling caused significant increases in microglial survival and migration (Cheng et al., 2018). More studies with better-characterized reagents are needed.

#### TREM2 as Drug Target: Challenges

Besides directing attention to microglia as an approach to AD drug discovery, TREM2 itself may have promise as a target if the appropriate pharmacologic modulation can be identified. As mutants that increase AD risk seem to have a partial loss of TREM2 function, and considering that overexpression studies with TREM2 seem to enhance its function in processes associated with AD (Takahashi et al., 2005; Lee et al., 2018), one hypothesis is that augmenting the amount or function of TREM2 might oppose AD progression. However, increasing the function of a protein is challenging. Antibodies stabilizing TREM2 on the cell surface might be another possibility, including those that increase cell-surface TREM2 by blocking its proteolytic cleavage and release (Schlepckow et al., 2017; Thornton et al., 2017). Small molecule binders that stabilize TREM2 by blocking proteolytic cleavage or other mechanisms are conceivable but likely quite difficult to find. More promising, but further off, would be modulation of TREM2 regulatory factors. Such signaling or regulatory mechanisms specific enough to TREM2 to expect avoidance of undesired pleiotropic effects have not been reported. These may be revealed by ongoing research, including phenotypic screens for genes or compounds that boost TREM2 function. But as a caveat to this whole strategy, recall that TREM2 mutants that increase AD risk are quite rare. The vast majority of AD patients carry the common variant sequence of TREM2. There is no direct evidence in humans, and only limited evidence in model systems, that boosting the function of “normal” TREM2 would have beneficial effects for most AD patients. There is much to be learned about TREM2 and its promise as a drug target.

## Conclusions

To date, no disease-modifying treatment for AD has achieved Food and Drug Administration (FDA) approval despite many studies showing their efficacy in mouse models. Aβ and tau are key features of AD, and either could be a “disease-modifying” target in principle. Multiple failures to stop AD by facilitating Aβ clearance or inhibition of production have considerably increased the interest in other modalities to target this devastating disease, such as neuroinflammation.

Microglia were first recognized as key players in neurodegenerative diseases more than two decades ago. More recently, unambiguous genetic evidence clearly links microglia function to AD pathogenesis. A large percentage of disease-associated SNPs are present in genes that are specifically expressed in microglia, a finding that has boosted the interest in understanding how microglia could be targeted to treat AD. Accordingly, numerous microglia proteins or pathways have been suggested to be drug targets. Here we reviewed several target candidates and their drugability challenges. Ideal drug targets should have a strong biological case, ideally combining genetic evidence with a well-understood mechanism of action that is supported by *in vitro* and *in vivo* data. However, even targets which fullfill this criteria need to be evaluated on whether they can be drugged safely. For the targets that have been briefly discussed in this review, these high hurdles have not yet been overcome. We remain optimistic that microglia-driven mechanisms will eventually bear clinical success and that one of the many discussed targets in this article will find a drug in the market helping AD patients.

## Author Contributions

KB, AB, BC, JP, MR, RS, RT, TM wrote the manuscript. KB, RT, and TM edited the manuscript.

## Conflict of Interest Statement

JP, RT, and TM are full-time employees of the Abbvie Foundational Neuroscience Center, Cambridge, MA, US. KB and MR are or have been full-time employees of AbbVie Deutschland GmbH & Co. KG, Ludwigshafen, Germany at the time of writing this article,. AB is a full-time employee of Janssen Research & Development LLC, San Diego, CA, US. BC is a full-time employee of Sage Therapeutics, Cambridge, MA, US. RS is a full-time employee of Paracelsus Neuroscience, Metuchen, NJ, US.
